# Supplementary material for: Glycerol 3-phosphate phosphatase/PGPH-2 counters metabolic stress and promotes healthy aging via a glycogen sensing-AMPK-HLH-30-autophagy axis in C. elegans
Source: Nat Commun. 2023 Aug 25;14:5214. doi: 10.1038/s41467-023-40857-y (PMC10457390; doi:10.1038/s41467-023-40857-y)
Supplement: Supplementary file 1 — Supplementary Information [file 41467_2023_40857_MOESM1_ESM.pdf]

Supplementary information

Glycerol 3-phosphate phosphatase/PGPH-2 counters metabolic stress and promotes healthy aging via a glycogen sensing-AMPK-HLH-30-autophagy axis in *C. elegans*

Possik, et al.,

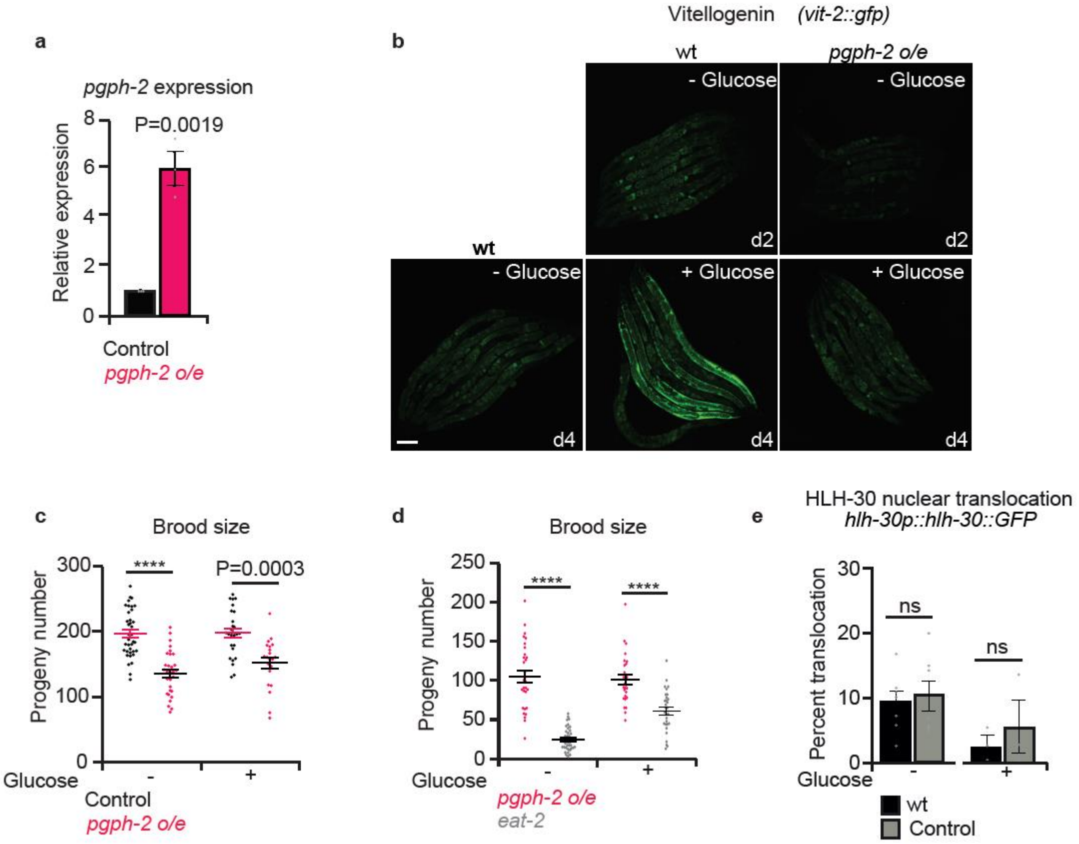

Figure S1. Related to figure 1. For all figure panels, **** represents *P<0.0001*. **a** Relative expression of *pgph-2* in control and *pgph-2 o/e* animals synchronized young adult animals. Data represent mean ± SEM, *n* = 3 independent experiments. *P* values were obtained using Student's two-tailed *t*-test. **b** Representative confocal images showing yolk distribution in WT and *pgph-2* o/e animals expressing the VIT-2::GFP in normal growth conditions or plates supplemented with 2% glucose at indicated age (d2= day 2, d4= day 4). Scale bar = 100 µm. **c-d** Brood size as measured by the number of progeny per animal in indicated strains grown on normal growth medium or plates supplemented with 2% glucose. Data represent mean ± SEM, *n* = 3 independent experiments. *P* values were obtained using one-way ANOVA with Bonferroni correction. **e** HLH-30 nuclear translocation in WT and control animals expressing the HLH-30::GFP transgene and grown in normal growth conditions or plates supplemented with 2% glucose. Data represent mean ± SEM from four independent experiments. *P* values were obtained using one-way ANOVA with Bonferroni correction.

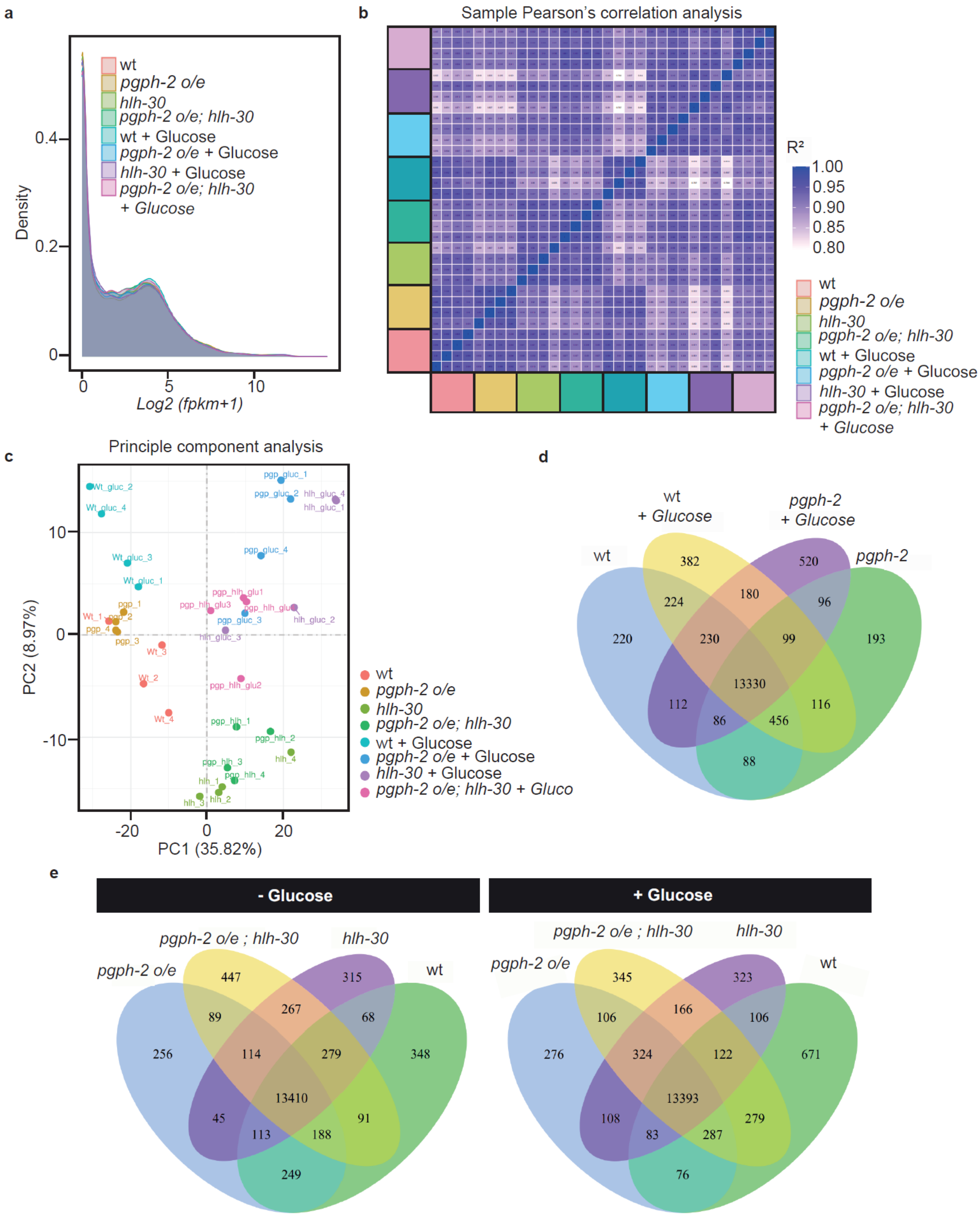

Figure S2. Related to figure 3. a Sample distribution of fragments per kilobase of exon per million mapped fragments (FPKM). **b** Pairwise Pearson's correlation analysis of eight samples with four biological replicates. **c** Sample 2D principle component analysis of the transcriptomic variation: PC1 (35.82%), PC2 (8.97 %). **d-e** Venn diagrams showing co-expression of genes between indicated groups.

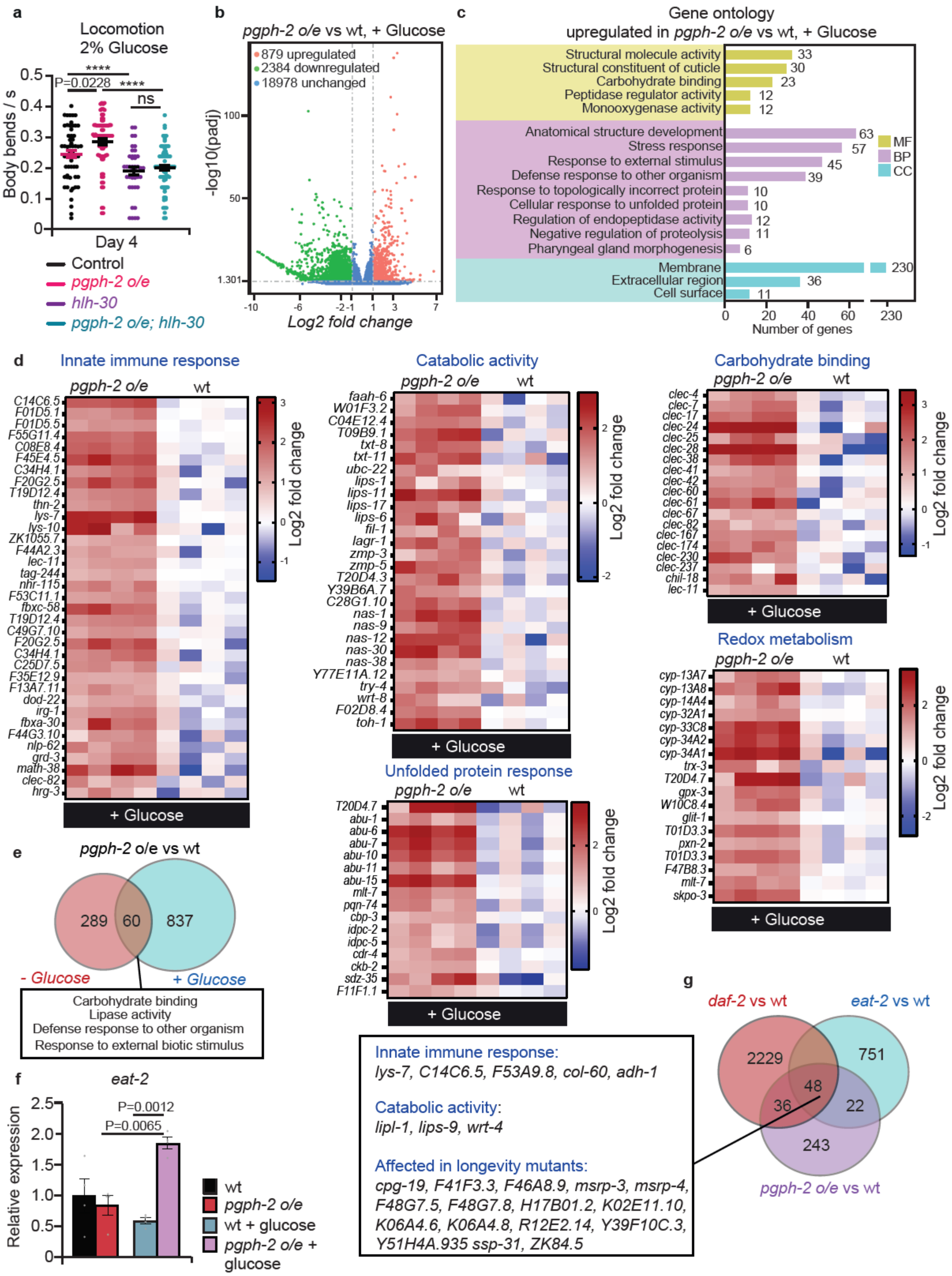
a
Locomotion
2% Glucose
Body bends / s
P=0.0228
****
****
ns
Day 4
Control
pgph-2 o/e
hlh-30
pgph-2 o/e; hlh-30
b
pgph-2 o/e vs wt, + Glucose
879 upregulated
2384 downregulated
18978 unchanged
-log10(padj)
Log2 fold change
c
Gene ontology
upregulated in pgph-2 o/e vs wt, + Glucose
Structural molecule activity 33
Structural constituent of cuticle 30
Carbohydrate binding 23
Peptidase regulator activity 12
Monooxygenase activity 12
Anatomical structure development 63
Stress response 57
Response to external stimulus 45
Defense response to other organism 39
Response to topologically incorrect protein 10
Cellular response to unfolded protein 10
Regulation of endopeptidase activity 12
Negative regulation of proteolysis 11
Pharyngeal gland morphogenesis 6
Membrane 230
Extracellular region 36
Cell surface 11
MF
BP
CC
Number of genes
d
Innate immune response
Catabolic activity
Carbohydrate binding
Redox metabolism
Unfolded protein response
pgph-2 o/e
wt
Log2 fold change
+ Glucose
e
pgph-2 o/e vs wt
289
60
837
- Glucose
+ Glucose
Carbohydrate binding
Lipase activity
Defense response to other organism
Response to external biotic stimulus
f
eat-2
Relative expression
P=0.0012
P=0.0065
wt
pgph-2 o/e
wt + glucose
pgph-2 o/e + glucose
g
daf-2 vs wt
eat-2 vs wt
2229
751
48
36
22
243
pgph-2 o/e vs wt
Innate immune response:
lys-7, C14C6.5, F53A9.8, col-60, adh-1
Catabolic activity:
lipl-1, lips-9, wrt-4
Affected in longevity mutants:
cpg-19, F41F3.3, F46A8.9, msrp-3, msrp-4, F48G7.5, F48G7.8, H17B01.2, K02E11.10, K06A4.6, K06A4.8, R12E2.14, Y39F10C.3, Y51H4A.935 ssp-31, ZK84.5

Figure S3. Related to figure 3. a Locomotion analysis on day 4 of age, measured by body bends per second in control, *pgph-2 o/e*, *pgph-2 o/e; hlh-30 (tm1978)*, and *hlh-30 (tm1978)* strains grown on plates supplemented with 2% glucose. Data represent mean ± SEM from three independent experiments. *P* values are obtained by one-way ANOVA with the Bonferroni correction. **** represents *P<0.0001*. **b** Volcano plots showing differentially expressed genes in WT vs *pgph-2 o/e* animals on plates supplemented with 2% glucose. Genes with a fold change > 2 and a *P* value smaller than 0.05 was considered significantly changed. Red, green, and blue indicate genes that are significantly upregulated, significantly downregulated, or not significantly changed, respectively. **c** Gene ontology (GO) annotations of genes significantly upregulated in *pgph-2 o/e* animals in comparison to WT grown on plates supplemented with 2% glucose and belonging to the molecular function (MF), biological processes (BP), and cellular component (CC) categories. **d** Heat map representing the differential expression of selected genes in WT and *pgph-2 o/e* animals grown on plates supplemented with 2% glucose and belonging to the carbohydrate binding, innate immune response, catabolic activity, unfolded protein response and ROS detoxification categories. **e** Venn diagram showing the number of genes upregulated in *pgph-2 o/e* animals vs WT simultaneously in normal growth conditions and 2% glucose excess conditions. **f** Relative gene expression of *eat-2* in WT and *pgph-2 o/e* animals in normal growth and excess glucose conditions. Data is denoted as mean ±SEM and is retrieved from RNA-seq results with four biological replicates per condition. *P* values were obtained using one-way ANOVA with Bonferroni correction. **g** Venn Diagram showing the number of genes upregulated simultaneously in *pgph-2 o/e* animals and the longevity mutants *daf-2(e1370)* and *eat-2(ad465)*.

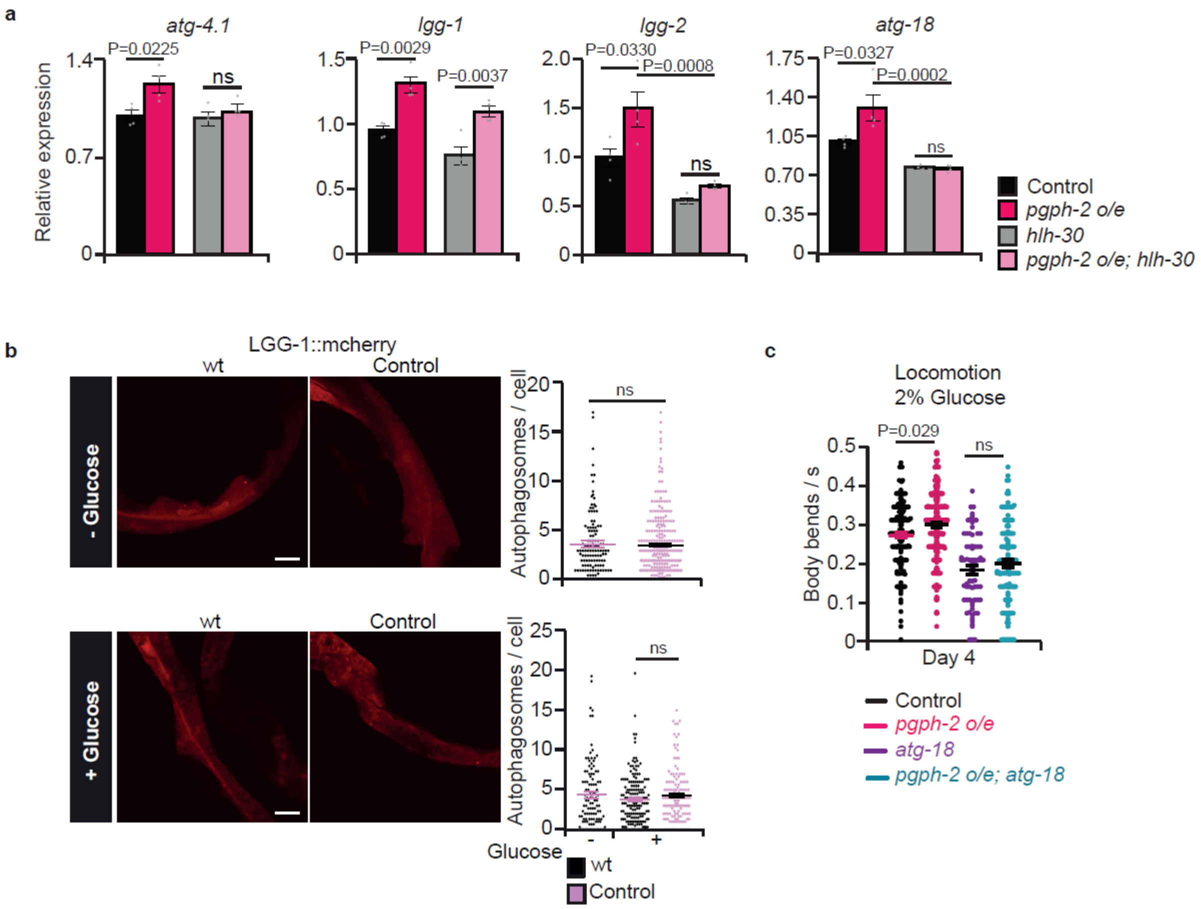

Figure **S4. Related to figure 4. a** Relative expression of indicated genes autophagy genes in WT, *pgph-2 o/e*, *pgph-2 o/e; hlh-30* and *hlh-30* mutant animals. Data is plotted as mean ±SEM and is retrieved from RNA-seq results with four biological replicates per sample. *P* values were obtained using one-way ANOVA with Bonferroni correction. **b** Representative confocal images and quantification of LGG-1::mCherry puncta in the intestines of day 1 WT and control animals under normal growth condition and glucose excess conditions. Data is shown using dot plots with denoted mean ± SEM from three independent experiments. *P* values are obtained by unpaired two-sided Student's t-test for normal conditions and one-way ANOVA with Bonferroni correction for 2% glucose conditions. **c.** Locomotion analysis on day 4 of age, measured by body bends per second control, *pgph-2 o/e*, *pgph-2 o/e; atg-18 (gk378)*, and *atg-18 (gk378)* strains grown on plates supplemented with 2% glucose. Data represent mean ± SEM from three independent experiments. *P* values are obtained by one-way ANOVA with the Bonferroni correction.

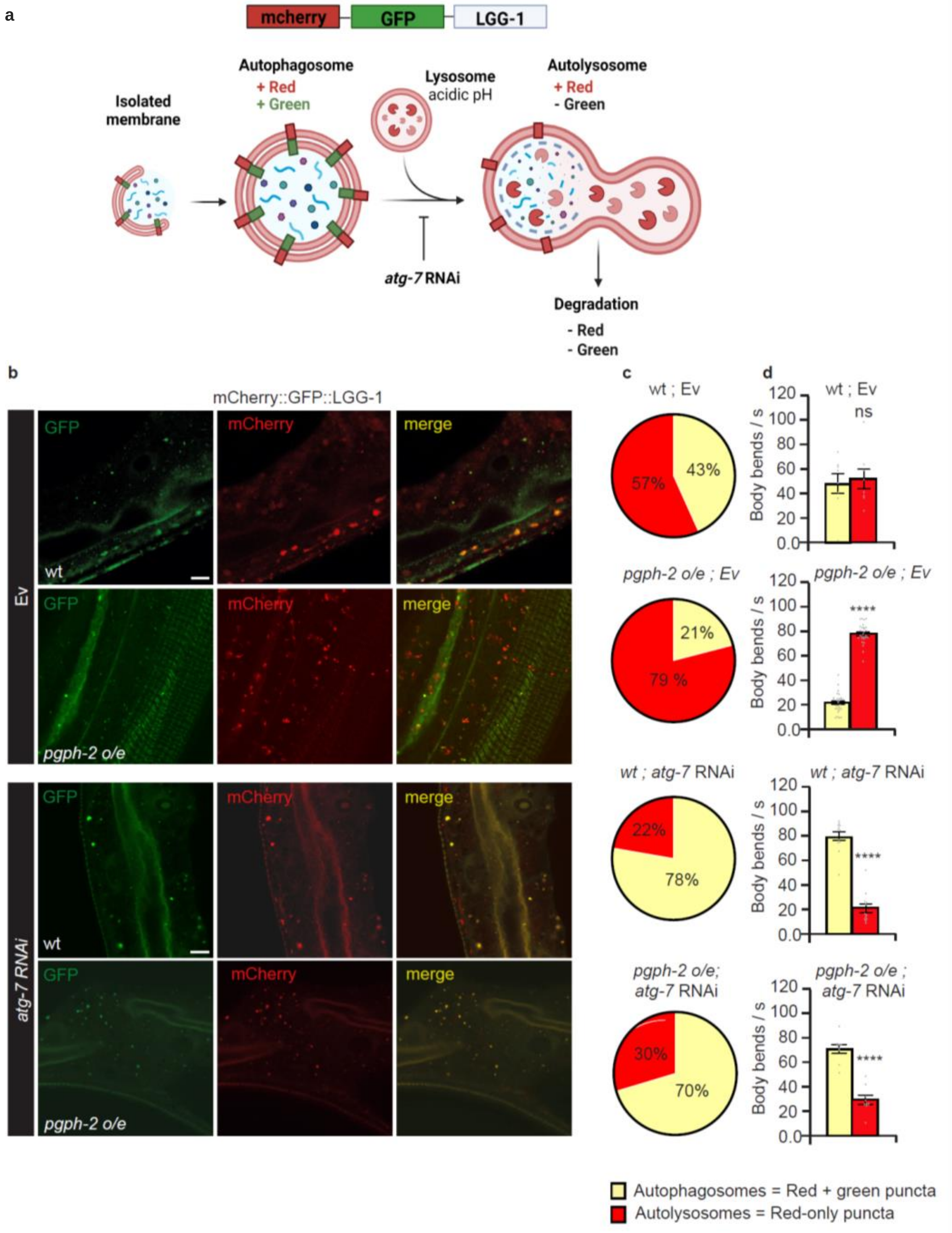

a
mcherry
GFP
LGG-1
Isolated membrane
Autophagosome
+ Red
+ Green
Lysosome
acidic pH
Autolysosome
+ Red
- Green
atg-7 RNAi
Degradation
- Red
- Green
b
mCherry::GFP::LGG-1
Ev
GFP
mCherry
merge
wt
pgph-2 o/e
atg-7 RNAi
c
wt ; Ev
43%
57%
pgph-2 o/e ; Ev
21%
79 %
wt ; atg-7 RNAi
22%
78%
pgph-2 o/e; atg-7 RNAi
30%
70%
d
Body bends / s
ns
****
Autophagosomes = Red + green puncta
Autolysosomes = Red-only puncta

Figure S5. Related to figure 4. a Schematic representation of the mCherry::GFP::LGG-1 fluorescence states in the autophagic pathway enabling the distinction between autophagosomes (red and green co-localization) and autolysosomes (red-only puncta). Scheme created using Biorender.com. **b** Representative confocal images of intestinal sections in day 1 adult WT and *pgph-2 o/e* transgenic animals expressing the mCherry::GFP::LGG-1 transgene and grown on EV or *atg-7* RNAi. The scale bar = 5 µm. **c, d** Pie charts and quantitative bar graphs **(d)** indicating the percentage of autophagosomes and autolysosomes counted in intestinal confocal images of one day adult WT *and pgph-2 o/e* transgenic animals expressing the mCherry::GFP::LGG-1 transgene and grown on EV or *atg-7* RNAi. Data denotes mean ± SEM from multiple animals imaged in three independent experiments and *P* values were obtained using unpaired two-sided Student's *t*-test. **** represents *P<0.0001*. N numbers are shown in datasource.

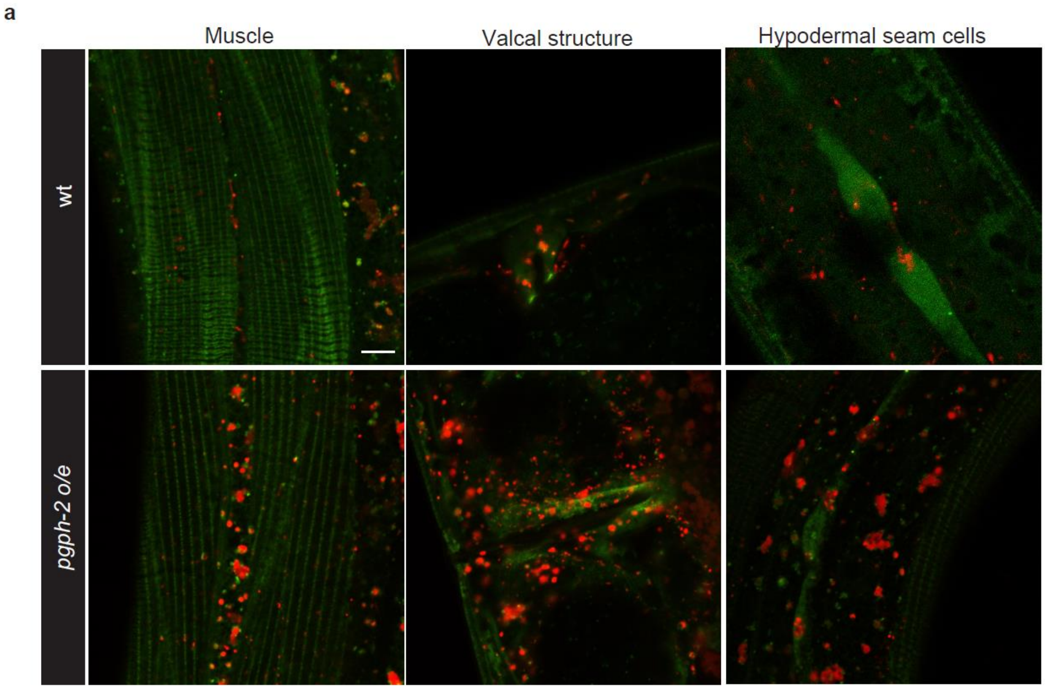

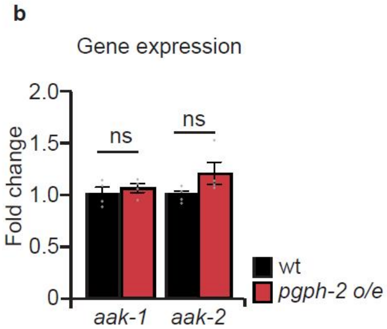

Figure S6. Related to figure 4 and 5. a Representative confocal images of muscle, valval structure and hypodermis in one day adult WT and *pgph-2 o/e* transgenic animals expressing the mCherry::GFP::LGG-1 transgene on normal growth medium. The scale bar = 5 µm. **b** Relative expression of *aak-1* and *aak-2* genes in WT and *pgph-2 o/e* animals in normal conditions. Data represents mean ± SEM and is retrieved from RNA-seq results with four biological replicates per sample. *P* values were obtained using one-sided unpaired student's t-test.

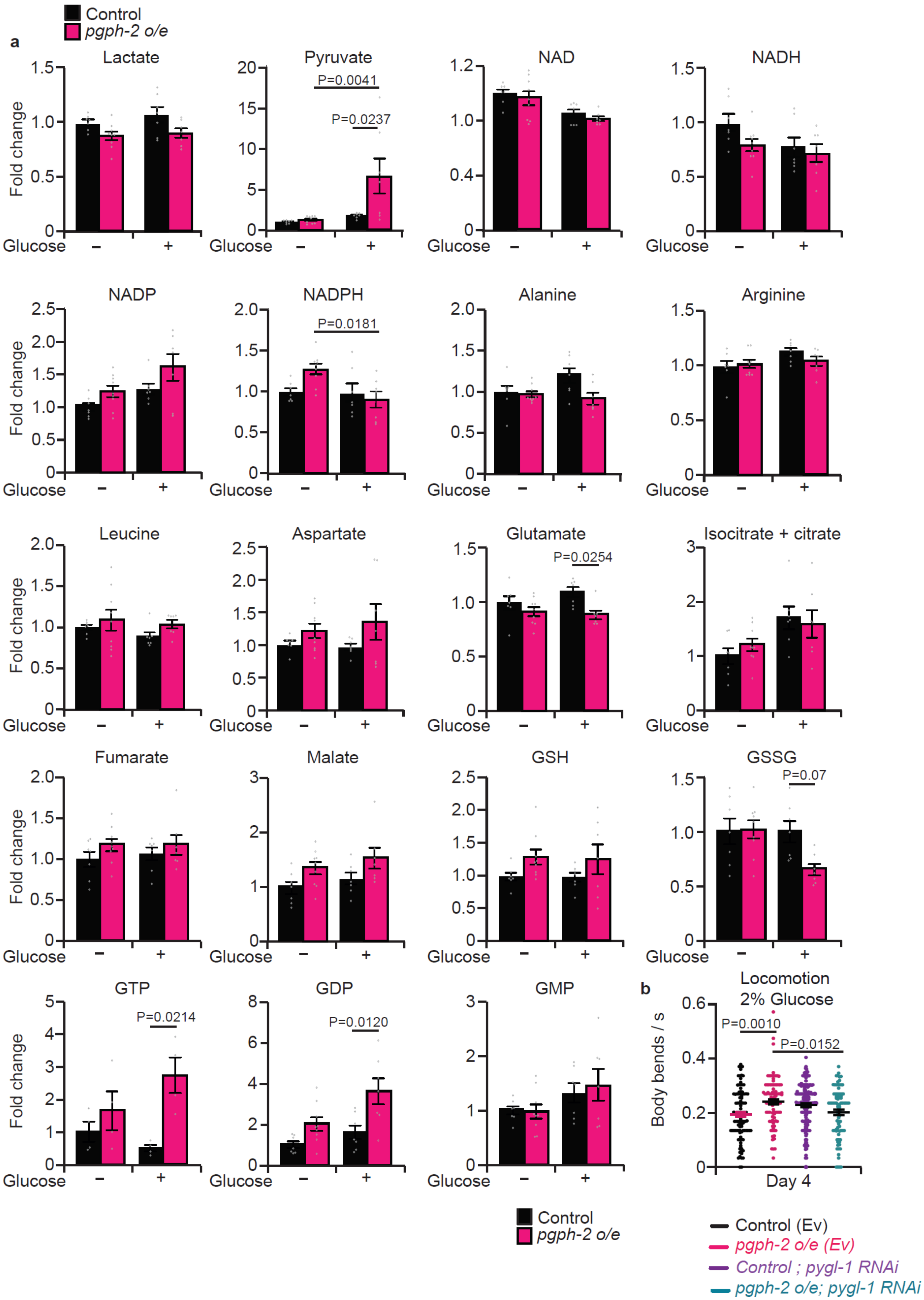

Control
pgph-2 o/e
a
Lactate
Pyruvate
P=0.0041
P=0.0237
NAD
NADH
NADP
NADPH
P=0.0181
Alanine
Arginine
Leucine
Aspartate
Glutamate
P=0.0254
Isocitrate + citrate
Fumarate
Malate
GSH
GSSG
P=0.07
GTP
P=0.0214
GDP
P=0.0120
GMP
Fold change
Glucose
b
Locomotion
2% Glucose
P=0.0010
P=0.0152
Body bends / s
Day 4
Control (Ev)
pgph-2 o/e (Ev)
Control ; pygl-1 RNAi
pgph-2 o/e; pygl-1 RNAi

Figure S7. Related to figure 6 and 7. a Relative metabolite levels and ratios of some metabolites in synchronized L4/young adult control and *pgph-2 o/e* animals grown on NGM plates or plates supplemented with 2% glucose. Data represent mean ± SEM from two independent experiments (control; n=7, *pgph-2 o/e;* n=9, control - 2% Glucose; n=7, *pgph-2 o/e* - 2% Glucose*;* n=7). *P* values are obtained by one-way ANOVA with the Bonferroni correction. Exact *P* values are shown in Supplementary Table 14 and are represented in figures as: *$P<0.05$; **$P<0.01$. **b** Locomotion analysis on day 4 of age, measured by body bends per second control, *pgph-2 o/e* strains treated with EV or *pygl-1* RNAi and grown on plates supplemented with 2% glucose. Data represent mean ± SEM from three independent experiments. *P* values are obtained by one-way ANOVA with the Bonferroni correction.
