# Supplementary material for: Glycerol 3-phosphate phosphatase/PGPH-2 counters metabolic stress and promotes healthy aging via a glycogen sensing-AMPK-HLH-30-autophagy axis in C. elegans
Source: Nat Commun. 2023 Aug 25;14:5214. doi: 10.1038/s41467-023-40857-y (PMC10457390; doi:10.1038/s41467-023-40857-y)
Supplement: Supplementary file 3 — Description of Additional Supplementary Files [file 41467_2023_40857_MOESM3_ESM.pdf]

Description of Additional Supplementary Files

Supplementary Data:

Supplementary Data 1: Summary results and statistical analysis of glucotoxicity assays.

Supplementary Data 2: List of genes upregulated in pgph-2 o/e animals vs WT in normal growth conditions.

Supplementary Data 3: Gene ontology terms describing genes upregulated in pgph-2 o/e aimals in comparison to WT in normal growth conditions.

Supplementary Data 4: List of genes upregulated in pgph-2 o/e animals vs WT in glucose excess conditions.

Supplementary Data 5: Gene ontology terms describing genes upregulated in pgph-2 o/e animals in comparison to WT in glucose excess conditions.

Supplementary Data 6: List of genes upregulated in pgph-2 o/e in comparison to WT simultaneously under normal and glucose excess conditions.

Supplementary Data 7: List of genes commonly upregulated in pgph-2 o/e animals, daf-2(e1370), and eat-2(ad465) mutant animals in comparison to WT.

Supplementary Data 8: List of the 214 HLH-30-dependent genes of 349 upregulated in pgph-2 o/e animals vs WT and downregulated in pgph-2 o/e; hlh-30 vs pgph-2 o/e

Supplementary Data 9: G profiler GO analysis for HLH-30-dependent genes, upregulated in pgph-2 o/e vs wt and downregulated in pgph-2o/e;hlh-30 vs pgph-2 o/e.

Supplementary Data 10: Autophagy gene expression fold change in WT, pgph-2 o/e, pgph-2 o/e; hlh-30 and hlh-30 animals.

Supplementary Data 11: C.elegans strains used in this study.

Supplementary Data 12: Table showing software names and versions used for RNA-seq analysis
