# Supplementary material for: Glycerol 3-phosphate phosphatase/PGPH-2 counters metabolic stress and promotes healthy aging via a glycogen sensing-AMPK-HLH-30-autophagy axis in C. elegans
Source: Nat Commun. 2023 Aug 25;14:5214. doi: 10.1038/s41467-023-40857-y (PMC10457390; doi:10.1038/s41467-023-40857-y)
Supplement: Supplementary file 16 — Reporting Summary [file 41467_2023_40857_MOESM16_ESM.pdf]

NCOMMS-22-47059A
Corresponding author(s): Elite Possik and Marc Prentki
Last updated by author(s): 25 July 2023

Reporting Summary

Nature Portfolio wishes to improve the reproducibility of the work that we publish. This form provides structure for consistency and transparency in reporting. For further information on Nature Portfolio policies, see our Editorial Policies and the Editorial Policy Checklist.

Statistics

For all statistical analyses, confirm that the following items are present in the figure legend, table legend, main text, or Methods section.

n/a	Confirmed	
☐	☒	The exact sample size (*n*) for each experimental group/condition, given as a discrete number and unit of measurement
☐	☒	A statement on whether measurements were taken from distinct samples or whether the same sample was measured repeatedly
☐	☒	The statistical test(s) used AND whether they are one- or two-sided<br>*Only common tests should be described solely by name; describe more complex techniques in the Methods section.*
☒	☐	A description of all covariates tested
☐	☒	A description of any assumptions or corrections, such as tests of normality and adjustment for multiple comparisons
☐	☒	A full description of the statistical parameters including central tendency (e.g. means) or other basic estimates (e.g. regression coefficient) AND variation (e.g. standard deviation) or associated estimates of uncertainty (e.g. confidence intervals)
☐	☒	For null hypothesis testing, the test statistic (e.g. *F*, *t*, *r*) with confidence intervals, effect sizes, degrees of freedom and *P* value noted<br>*Give P values as exact values whenever suitable.*
☒	☐	For Bayesian analysis, information on the choice of priors and Markov chain Monte Carlo settings
☒	☐	For hierarchical and complex designs, identification of the appropriate level for tests and full reporting of outcomes
☐	☒	Estimates of effect sizes (e.g. Cohen's *d*, Pearson's *r*), indicating how they were calculated

Our web collection on statistics for biologists contains articles on many of the points above.

Software and code

Policy information about availability of computer code

Data collection	Wormlab software from MBF Biosciences, version 2018, Leica TCS SP5, Histat (2.0.5), Stringtie (1.3.3b), Feature counts (1.5.0-p3)
Data analysis	Prism Graph pad versions 6 and 9, DESeq2 (1.20.0), edgeR (3.22.5), g:profiler, Image J. (1.52 a), Fiji win-64

For manuscripts utilizing custom algorithms or software that are central to the research but not yet described in published literature, software must be made available to editors and reviewers. We strongly encourage code deposition in a community repository (e.g. GitHub). See the Nature Portfolio guidelines for submitting code & software for further information.

Data

Policy information about availability of data

All manuscripts must include a data availability statement. This statement should provide the following information, where applicable:

- Accession codes, unique identifiers, or web links for publicly available datasets
- A description of any restrictions on data availability
- For clinical datasets or third party data, please ensure that the statement adheres to our policy

All relevant data generated or analyzed during this study are included in this manuscript and/or its supplementary information and datasource or can be obtained from the

corresponding authors upon request. The data underlying Figs. 1-8 and supplementary Figs. 1-7 are provided as Source data. The metabolomics data is now publically available at Metabolights database (MTBLS6847) and the RNA-seq at GEO (GSE228784) https://www.ncbi.nlm.nih.gov/geo/query/acc.cgi?acc=GSE228784.

Human research participants

Policy information about studies involving human research participants and Sex and Gender in Research.

Reporting on sex and gender	Not applicable
Population characteristics	Not applicable
Recruitment	Not applicable
Ethics oversight	Not applicable

Note that full information on the approval of the study protocol must also be provided in the manuscript.

Field-specific reporting

Please select the one below that is the best fit for your research. If you are not sure, read the appropriate sections before making your selection.

[X] Life sciences [ ] Behavioural & social sciences [ ] Ecological, evolutionary & environmental sciences

For a reference copy of the document with all sections, see nature.com/documents/nr-reporting-summary-flat.pdf

Life sciences study design

All studies must disclose on these points even when the disclosure is negative.

Sample size	The sample sizes are indicated in supplementary tables and legends and clearly shown in datasource. Sample size here is conform to most C.elegans research and publications. Lifespan and survival assays, 90 animals per group per independent repeat. Biochemical studies, pools of thousands of worms pelleted to about 250 microliters. Details in the manuscript. Examples: Schultz et al., 2007 Cell metabolism, Lapierre et al., 2013 Nature Communications, Weir et al., 2017 Cell metabolism, Visviskis et al., 2017 Immunity, Possik et al., 2022, Nature Communications. For RNA-Seq, power analysis indicated in methods was performed.
Data exclusions	No data were excluded in this study
Replication	All the experiments have been performed at least three independent times unless noted otherwise. Number of biological replicates and independent repeats is detailed in the supplementary tables and legends.
Randomization	Worms were randomly picked for experiments where sample size is below 150 animals for most independent repeats. For biochemical studies, pools of thousands of worms have been collected and analyzed with at least 4 biological replicates unless noted otherwise. Randomization like in clinical trials or experiments with rodents is not applicable as for all experiments, picking worms from plates with thousands of worms is on its own a random event. Visually, from pools of worms that look identical, the selection is random.
Blinding	We did not perform blinding allocations. However, most key experiments have been performed by independent lab members and trainees and the results obtained confirmed the statements of the manuscript. Moreover, quantifications of autophagy and analysis have been performed independently by at least 2 independent researchers and data was averaged.

Behavioural & social sciences study design

All studies must disclose on these points even when the disclosure is negative.

Study description	Not applicable
Research sample	Not applicable
Sampling strategy	Not applicable
Data collection	Not applicable

Data collection	Not applicable
Timing	Not applicable
Data exclusions	Not applicable
Non-participation	Not applicable
Randomization	Not applicable

Ecological, evolutionary & environmental sciences study design

All studies must disclose on these points even when the disclosure is negative.

Study description	Not applicable
Research sample	Not applicable
Sampling strategy	Not applicable
Data collection	Not applicable
Timing and spatial scale	Not applicable
Data exclusions	Not applicable
Reproducibility	Not applicable
Randomization	Not applicable
Blinding	Not applicable

Did the study involve field work? ☐ Yes ☒ No

Field work, collection and transport

Field conditions	Not applicable
Location	Not applicable
Access & import/export	Not applicable
Disturbance	Not applicable

Reporting for specific materials, systems and methods

We require information from authors about some types of materials, experimental systems and methods used in many studies. Here, indicate whether each material, system or method listed is relevant to your study. If you are not sure if a list item applies to your research, read the appropriate section before selecting a response.

Materials & experimental systems

n/a	Involved in the study
☐	☒ Antibodies
☒	☐ Eukaryotic cell lines
☒	☐ Palaeontology and archaeology
☐	☒ Animals and other organisms
☒	☐ Clinical data
☒	☐ Dual use research of concern

Methods

n/a	Involved in the study
☒	☐ ChIP-seq
☒	☐ Flow cytometry
☒	☐ MRI-based neuroimaging

Antibodies

Antibodies used	pAMPKα (T172) rabbit mAb from Cell signalling (1 :1000 dilution prepared in TBS-T1x + 5% BSA + 50uM NaF and incubated overnight at 4° C). The α-tubulin antibody is from Abcam (1:10000 dilution prepared in 5% milk in TBST1X).
Validation	The p-AMPK (Thr172) antibody and Tubulin have been used by multiple C.elegans groups in various high impact publications including our previous work (Schultz et al., 2007, Cell metabolism; Cabreiro et al., 2013; Cell, Weir et al., 2017, Cell metabolism). This has been very well established.

Animals and other research organisms

Policy information about studies involving animals; ARRIVE guidelines recommended for reporting animal research, and Sex and Gender in Research

Laboratory animals	*Caenorhabditis elegans nematodes (C.elegans)*
Wild animals	The animals used were obtained from CGC and were not captured in the Wild.
Reporting on sex	The animals are either hermaphrodites or males. In all experiments with used hermaphrodites. Males were only used for genetic crossing. Sex analysis is not applicable.
Field-collected samples	The study did not involve samples collected in the field
Ethics oversight	Not applicable

Note that full information on the approval of the study protocol must also be provided in the manuscript.
